# Diversity of apostome ciliates, *Chromidina* spp. (Oligohymenophorea, Opalinopsidae), parasites of cephalopods of the Mediterranean Sea

**DOI:** 10.1051/parasite/2016033

**Published:** 2016-08-17

**Authors:** Dhikra Souidenne, Isabelle Florent, Marc Dellinger, Jean Lou Justine, Mohamed Salah Romdhane, Hidetaka Furuya, Philippe Grellier

**Affiliations:** 1 UMR 7245 CNRS MCAM, Muséum National d’Histoire Naturelle, Sorbonne Universités CP 52, 57 rue Cuvier 75005 Paris France; 2 UR03AGRO1 Ecosystèmes et Ressources Aquatiques, Institut National Agronomique de Tunisie 43 avenue Charles Nicolle 1082 Tunis Tunisia; 3 ISYEB, Institut de Systématique, Évolution, Biodiversité (UMR7205 CNRS, EPHE, MNHN, UPMC), Muséum National d’Histoire Naturelle, Sorbonne Universités CP 51, 55 rue Buffon 75005 Paris France; 4 Department of Biology, Graduate School of Science, Osaka University 1-1 Machikaneyama 560-0043 Toyonaka, Osaka Japan

**Keywords:** Apostome, Ciliate, Cephalopods, Neohapantotype, *Chromidina elegans*, *Chromidina chattoni* n. sp.

## Abstract

*Chromidina* spp. are enigmatic apostome ciliates (Oligohymenophorea, Opalinopsidae) that parasitise the renal and pancreatic appendages of cephalopods. Only four species have been described, among which only three have been formally named. No DNA sequence has been reported so far. To investigate *Chromidina* spp. diversity, we sampled cephalopods in the Mediterranean Sea off Tunis, Tunisia, and identified two distinct *Chromidina* spp. in two different host species: *Loligo vulgaris* and *Sepia officinalis*. From haematoxylin-stained slides, we described morphological traits for these parasitic species and compared them to previous descriptions. We also re-described the morphology of *Chromidina elegans* (Foettinger, 1881) from Chatton and Lwoff’s original materials and designated a neohapantotype and paraneohapantotypes for this species. We describe a new species, *Chromidina chattoni* Souidenne, Florent and Grellier n. sp., found in *L. vulgaris* off Tunisia, and evidence for a probable novel species, found in *S. officinalis* off Tunisia, although this latter species presents similarities to some morphological stages previously described for *Chromidina cortezi* Hochberg, 1971. We amplified, for the first time, an 18S rDNA marker for these two *Chromidina* species. Phylogenetic analysis supports the association of *Chromidina* within apostome ciliates. Genetic distance analysis between 18S rDNA sequences of representative apostomes indicates *Pseudocollinia* as the most closely related genus to *Chromidina*.

## Abstract (fr)

Les *Chromidina* spp. sont des ciliés apostomes (Oligohymenophorea, Opalinopsidae) énigmatiques qui parasitent les sacs rénaux et pancréatiques des céphalopodes. Seules quatre espèces ont été décrites dont trois ont été formellement nommées. Aucune séquence d’ADN n’a été reportée à ce jour. Afin d’explorer la diversité des espèces du genre *Chromidina*, nous avons échantillonné des céphalopodes en mer Méditerranée, au large de Tunis en Tunisie, et avons identifié deux espèces distinctes de *Chromidina* spp. chez deux hôtes différents, *Loligo vulgaris* et *Sepia officinalis*. À partir de lames colorées à l’hématoxyline, nous avons analysé les traits morphologiques de ces parasites et les avons comparés aux descriptions antérieures. Nous avons également redécrit la morphologie de *Chromidina elegans* (Foettinger, 1881) à partir du matériel original de Chatton et Lwoff et avons désigné un néohapantotype et des paranéohapantotypes pour cette espèce. Nous décrivons une nouvelle espèce, *Chromidina chattoni* Souidenne, Florent and Grellier n. sp., trouvée chez *L. vulgaris* en Tunisie, et signalons une espèce probablement nouvelle trouvée chez *S. officinalis* en Tunisie, bien que cette dernière présente des similitudes avec des stades morphologiques précédemment décrits pour *Chromidina cortezi* Hochberg, 1971. Nous avons amplifié, pour la première fois, le marqueur ADNr 18S pour ces deux espèces de *Chromidina*. L’analyse phylogénétique supporte l’association des *Chromidina* au sein des ciliés apostomes. L’analyse des distances génétiques de ce marqueur chez des apostomes représentatifs suggère que le genre *Pseudocollinia* est le plus proche du genre *Chromidina*.

## Introduction

Species of *Chromidina* Gonder, 1905 [15] are enigmatic apostome ciliates being, after dicyemids, the most frequently encountered parasites inhabiting the reno-pancreatic appendages of cephalopods [18–20, 30]. Initially in 1881, Foettinger observed infusoria parasites in *Sepia elegans* renal appendages. Recognising that these parasites were atypical protozoa, he erected the genus *Benedenia* [11], named after the embryologist Edouard van Beneden. In 1905, Gonder re-examined these parasites, highlighting their nuclear system organised as a network throughout the cell. Since this type of nuclear system arrangement was considered by Gonder as typical of chromidial systems, he changed the genus name from *Benedenia* to *Chromidina* [11]. Currently, 23 cephalopod species have been recorded as hosts of *Chromidina* spp., of which only three have been formally described: *Chromidina elegans* (Foettinger, 1881), *C. coronata* (Foettinger, 1881) and *C. cortezi* Hochberg, 1971. A fourth has been described morphologically but not named (Table 1) [25]. These ciliates infest mainly pelagic cephalopods, such as epi-meso pelagic squids, cuttlefishes and octopuses. Occasionally, they may be found in the kidneys of benthic hosts, if these hosts include a young nektonic stage during their development [13].

**Table 1.** Summary of morphological features of *Chromidina* species.

Parasite	Host		Localisation		Specimens and status	Aspect of the anterior end (apex)	Crown of cilia	Presence of apotomites	Presence of hypertrophont	Presence of tomites	Number of kineties	18S rDNA sequence	Reference
Species	Type	Other hosts described	Type	Others									
*C. elegans*	*Sepia elegans*	*Sepia orbignyana, Illex coindetti, Todarodes sagittatus, Octopus salutii*	Naples, Italy	Mediterranean Sea, English Channel and Banyuls-sur-Mer, France	MNHN-IR-1970-9 (neohapantotype) MNHN-IR-1970-18 to 64 (paraneohapantotypes)	Club-like	No	ND	Yes	Yes	14	–	[4, 11, 15], this study
*C. coronata*	*Octopus vulgaris*	*Eledone cirrhosa, Sepiola rondeleti, Scaeurgus unicirrhus, Illex coindetti*	Naples, Italy	Mediterranean Sea and English Channel		Claviform	Yes	ND	ND	ND	ND	–	[11, 20]
*C. cortezi*	*Pterygioteuthis giardi*	–	Gulf of California	Gulf of Mexico		Pointed	No	Yes	Yes	Yes	12		[17]
*Chromidina* sp.	*Illex coindetti*	–	Gulf of Mexico	–		Bulbous	No	Yes	ND	Yes	12		[25]
*C. chattoni*	*Loligo vulgaris*	–	Tunis, Tunisia, Mediterranean Sea	–	MNHN-IR-2016-326 (hapantotype) MNHN-IR-2016-327-341 (parahapantotypes)	Globulous	No	ND	ND	Yes	13	LT546660 LT546661 LT546662	This study
*Chromidina* sp. S50	*Sepia officinalis*	–	Tunis, Tunisia, Mediterranean Sea	–	MNHN-IR-2016-108 (voucher)	Conical	No	ND	ND	ND	–	LT546663	This study

ND: not described.

*Chromidina* spp. have a polymorphic dixenous life cycle, with two different budding processes which are monotomy and palintomy. The adult stage, the vermiform tropho-tomont, has a maximum body length varying from 400 μm to 2,000 μm [20]. Some rare adult stages can have an accelerated growth process. Their size increases so quickly that their length can measure up to 5,000 μm. Given their unusual extended size, these adult stages are called hypertrophonts. The tropho-tomont is uniformly ciliated and has no cytostome [4]. It is attached through its anterior end to the host kidney tissues with its body bathing in the renal fluids, and feeds by nutriment absorption from host cells and fluids [25]. Division by monotomy produces a single long bud from the posterior end, the apotomite, which is morphologically similar to its parent and develops into a second generation of tropho-tomonts after detachment and colonisation of the host kidney. Division by palintomy produces smaller buds that form a typical chain of individuals attached to the tropho-tomont, which differentiate into tomites. Budding occurs only from the posterior end. The tomite is a small ciliate form with a unique ciliature and a cytostome [4]. When detached, it is believed that the tomite leaves the renal appendages to be released with passage of urine into the sea. This stage is presumed to encyst, as a phoront, and to infest an intermediate host [17, 18]. However, no intermediate host has been confirmed so far. Chatton and Lwoff [4] grouped *Chromidina* within the apostome ciliates even though their life cycle and their morphology show important differences from typical apostomes. The main argument for such an association is that *Chromidina* tomites share ciliature organisation similar to that of apostome tomites. Besides the reference work of Chatton and Lwoff [4], reports by Hochberg [17, 18, 20], and the recent description of a novel *Chromidina* sp. by Landers [25], little is known about this enigmatic genus and notably, no DNA sequence has been reported so far.

In the present study, we examined the Tunisian teuthofauna for infections by *Chromidina.* We report here the description of two *Chromidina* species and compare their morphological traits to those of previously described parasites. We provide, for the first time, *Chromidina* 18S rDNA sequences. In the course of this study, we also re-described *Chromidina elegans* and designated a neohapantotype and paraneohapantotypes from the Chatton and Lwoff original slide collection [4]. A phylogenetic analysis was performed to evaluate the association of *Chromidina* with apostomes within the Oligohymenophorea.

## Materials and methods

### Host sampling and isolation of parasites

Cephalopods were obtained from fishermen from the harbour of La Goulette, who collected them off Tunis, in the Mediterranean Sea (36°49′9.11″ N, 10°18′22.49″ E), in January 2013 and January 2014, using traditional earthenware jugs as fishing tools [31]. In total, 5 individuals of *Loligo vulgaris* and 38 individuals of *Sepia officinalis* were collected. The cephalopod species identification was based on morphological features relying on cited identification keys [23] and species descriptions (Marine Species Identification portal, http://species-identification.org). The hosts were rapidly dissected and their renal appendages collected. Small pieces of these renal appendages were smeared on glass microscope slides, which were immediately fixed in Bouin’s fluid for 24 h then stored in 70% ethanol. The smears were then stained in Ehrlich’s Haematoxylin and counterstained in eosin, then mounted with Entellan (Merck, Rahway, New Jersey) [12]. In parallel, additional renal appendage pieces from the same cephalopod host were put in individual Petri dishes in sterile distilled water and observed with a stereomicroscope to collect *Chromidina* specimens with Pasteur pipettes. These parasites were then transferred to new Petri dishes to be washed several times, in order to eliminate the host tissue possibly remaining attached to the parasites. Washed parasites were then transferred, one by one, to sterile Eppendorf tubes filled with 70% ethanol and were stored at 4 °C until use for DNA isolation.

### Morphological studies

Smears were observed by light microscope. Images were recorded using a Nikon DXM 1200C camera and processed using ImageJ software (http://imagej.nih.gov/ij/). Measurements were made with the aid of a micrometric slide by using the ImageJ Set Scale module. Target *Chromidina* spp. were observed on slides prepared from positive hosts; specifically, three infected *L. vulgaris* individuals (C21, C22 and C23) and one infected *S. officinalis* individual (S50). Smears of these specimens were deposited in the Protist collection of the Muséum National d’Histoire Naturelle, Paris, France (www.mnhn.fr/fr/collections/ensembles-collections/invertebres-marins/protistes). The morphologies of these *Chromidina* spp. specimens were compared to descriptions from the literature and to the original Chatton and Lwoff’s smeared slides used to describe *Chromidina elegans* [4], which are deposited in the Protist collection of the Muséum National d’Histoire Naturelle. Since no type slide related to Foettinger’s work [11] could be localised, we undertook to re-describe this species to which we associated a neohapantotype and paraneohapantotypes.

### Molecular studies

DNA extractions were performed using Chelex^TM^ (Biorad), following the methodology described in reference [9]. The 18S rDNA loci of *Chromidina* were amplified by using the universal primer pair MDP4 (forward, 5′-CTGGTTGATCCTGCCAG-3′, [1]) and MDP3 (reverse, 5′-GACGGGCGGTGTGTAC-3′, [26]), and two *Chromidina-*specific primers designed in the course of this study: FurF (forward, 5′-GCAGGCGCGTAAATTA-3′) and FurR (reverse, 5′-CACTCGAAATCGGTAGCA-3′). The HOT FIREPol^®^ DNA Polymerase enzyme was used as recommended by the supplier. Briefly, following an initial denaturation period of 12 min at 94 °C, 35 cycles of denaturation for 30 s at 94 °C, annealing for 1 min at 50 °C, and elongation for 2 min at 72 °C, were performed, and the PCR was terminated by a final elongation for 7 min at 72 °C. The quality of the yielded amplicons (single band, proper size) was validated after electrophoresis in 1% Agarose^TM^ gels in 0.5× TAE buffer, in the presence of 0.5 μg/mL ethidium bromide and UV illumination. The positive amplicons were either sequenced directly or purified using Illustra^TM^ GFX^TM^ PCR DNA and Gel Band Purification kit (GE Healthcare, France) to be cloned into pGEM^R^-T Easy vector (Promega, France) following the supplier’s recommendations. Positive clones were selected by PCR using universal T7 (5′-TAGTTATTGCTCAGCGGTGG-3′) and Sp6 (5′-ATTTAGGTGACACTATAG-3′) primers flanking the pGEM^R^-T Easy vector cloning site. DNA sequencing was performed by the Sanger method on PCR-amplified fragments, using appropriate primers (T7, Sp6, MDP4, MDP3, FurF, FurR) (Beckman Coulter Genomics, Takeley, UK). Raw chromatograms were analysed using the BioEdit 7.1.3.0 program [16] and loci were assembled using the MEGA 6.06 package [33]. The new 18S rDNA sequences (1516–1541 pb in length), obtained from *Chromidina* used for the morphological identification, were deposited in the EMBL database (LT46660–LT46663).

### Genetic distance between apostome species

18S rDNA sequences from the four *Chromidina* specimens isolated from the infected hosts and 10 18S rDNA sequences representative of each Apostomatia species, retrieved from the EMBL or GenBank databases, were aligned using the online version of MAFFT, version 7 (http://mafft.cbrc.jp/alignment/server/; [24]), using the secondary structure of RNA (Q-INS-I option). Evolutionary distances between the sequences were computed pairwise using p-distances and uniform rate analyses in MEGA 6.06 [33]. All ambiguous positions were removed for each sequence pair and there were a total of 1,477 positions in the final dataset. The numbers of base differences per site between sequences were expressed as percentages.

### Maximum likelihood and Bayesian rooted trees for Ciliophora

18S rDNA sequences from the four *Chromidina* specimens, and 25 sequences of representatives of Ciliophora and from the Dinoflagellate *Biecheleriopsis adriatica* (HG792067) taken as the outgroup, were aligned using the online version of MAFFT, version 7 (http://mafft.cbrc.jp/alignment/server/; [24]), using the secondary structure of RNA (Q-INS-I option), and the alignment was filtered out using the online version of GBlock [2]. GBlock settings were set to allow moderately strict flanking positions (Maximum number of contiguous non-conserved positions: 8; minimum length of a block: 10; no gap position allowed), yielding a confident alignment of 1,157 positions. A general time-reversible (GTR) substitution model with gamma-distributed rate variation across sites was suggested by JModeltest V2.1.3 as the best-fit model for this alignment [8]. Accordingly, a Bayesian phylogenetic tree was constructed with MrBayes v.3.2.3 [32], using lset nst = 6 rates = Invgamma Ngammacat = 4 parameters. Four simultaneous Monte Carlo Markov chains were run from random trees for a total of 5,000,000 generations in two parallel runs. A tree was sampled every 1,000 generations and 25% of the trees were discarded as “burn-in”. A consensus tree was constructed from the post-burn-in trees using FigTree v1.3.1, and posterior probabilities were calculated in MrBayes. In parallel, Maximum Likelihood analyses were performed using the same alignment and the GTR+G+I model, with MEGA 6.06 [33]. Bootstraps were estimated from 1,000 replicates.

Phylum: Ciliophora Doflein, 1901.

Subphylum: Intramacronucleata Lynn, 1996.

Class: Oligohymenophorea de Puytorac et al., 1974.

Subclass: Apostomatia Chatton & Lwoff, 1928.

Order: Astomatophorida Jankowksi, 1966.

Family: Opalinopsidae Hartog, 1906.

Genus: *Chromidina* Gonder, 1905.

## *Chromidina elegans* (Foettinger, 1881) Gonder, 1905 (Fig. 1)

Synonym: *Benedenia elegans* Foettinger, 1881.

**Figure 1.**
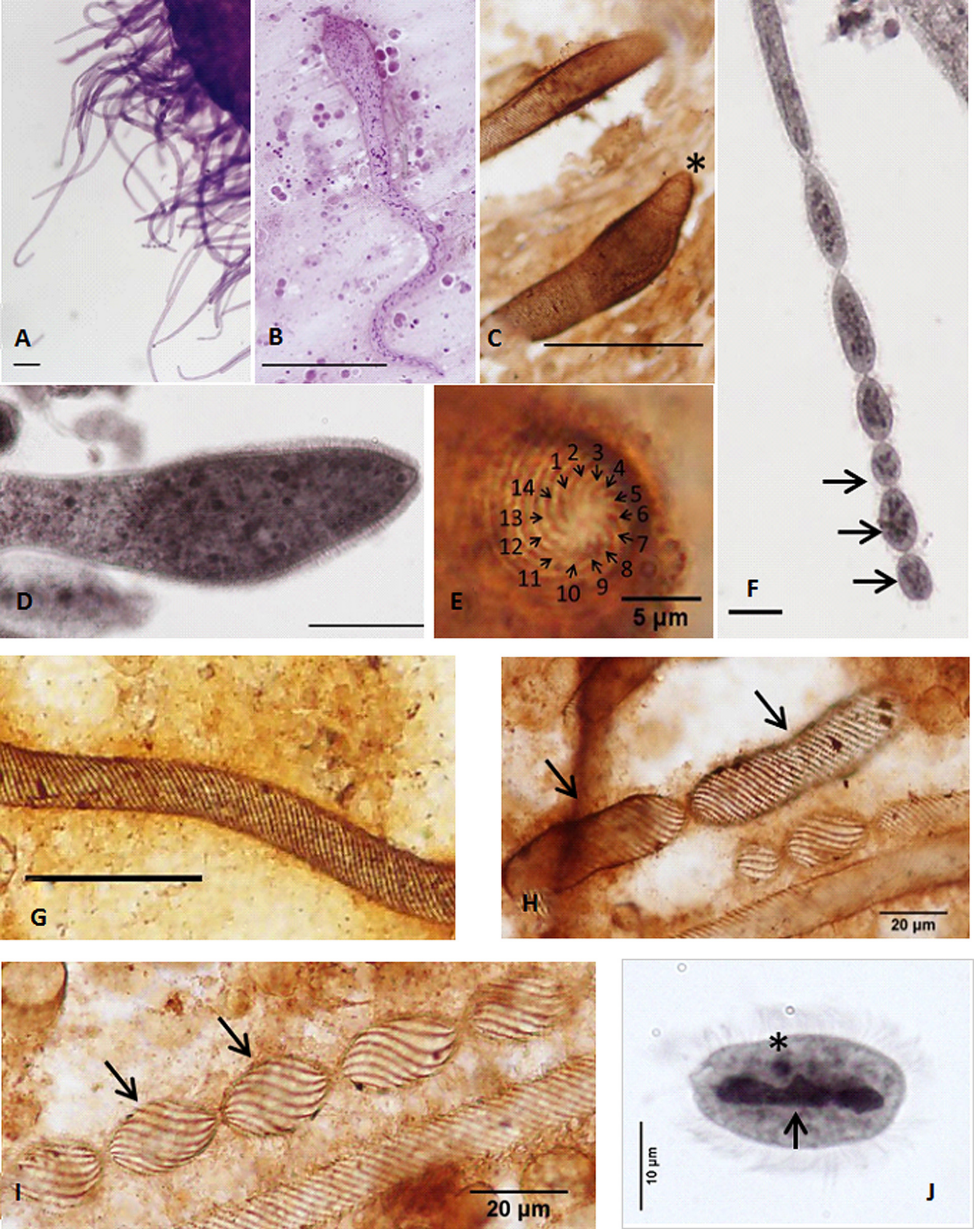
*Chromidina elegans* (Foettinger, 1881) Gonder, 1905: morphology. (A) General view of infected renal sac. Parasites are attached to the renal tissues by their anterior end with their cell body hanging free in the renal coelom. (B) Tropho-tomont. (C, D) Club-like and inflated anterior ends of tropho-tomonts (*: apical papillum). (E) Apical ciliature consisting of 14 kineties that extend continuously through the entire cell body. (F) Palintomy (arrow: protomites with condensed chromatin). (G) Cell body ciliature. (H) First generation of protomites (arrow: fission plan). (I) Chain of second generation of protomites (arrows). (J) Free tomite (*: micronucleus, arrow: macronucleus). (A, B) Haematoxylin staining. (C, E, G–I) Chatton’s silver impregnation. (D, F, J) Osmium staining. Unless otherwise indicated, bar = 100 μm. (A–J) Neohapantotype and paraneohapantotypes.

Host: *Sepia elegans* d’Orbigny, 1825.

Infection site: Renal appendages.

Other reported hosts: *Sepia orbignyana* Ferussac, 1826, *Illex coindetti* Vérany, 1837 in the Mediterranean Sea and English Channel; *Todarodes sagittatus* Lamarck, 1798 and *Octopus salutii* Vérany, 1839 in the Mediterranean Sea [4, 17, 29]. Wermel [34] observed *C. elegans*-like species in Russia on *Loligo* sp., and Jepps [22] and Clarke [6] in *Spirula spirula* Linnaeus, 1758 in the Atlantic Ocean.

Type material: Neohapantotype catalogued under No. MNHN-IR-1970-9 and paraneohapantotypes catalogued under Nos. MNHN-IR-1970-18 to 64 from Chatton and Lwoff’s work on *C. elegans* [4], deposited in the Protist Collection of the Muséum National d’Histoire Naturelle.

Type locality: Banyuls-sur-Mer, France (42°29′01″ N, 3°07′44″ E) [4].

Other reported localities: Initially described from Naples, Italy, by Foettinger [11].

Prevalence: No information available.

*Chromidina elegans* was initially described by Foettinger in 1881 but under the genus *Benedenia* Foettinger, 1881 and not *Chromidina* [11]. In his pioneering work, Foettinger did not designate a type species despite detailed descriptions of *C. elegans* (formerly *B. elegans*). We were unable to trace the existence of a corresponding type- or hapantotype-slide in the literature, and the Ciliate Resource Archive database (http://www.uoguelph.ca/~ciliates/) indicates that these are absent for this genus. Later, Chatton and Lwoff provided a detailed description of *C. elegans* and clearly designated in their monograph this species as the type species for the genus *Chromidina*, but without indicating any type- or hapantotype-slide [4]. However, Chatton’s slide collection was deposited by the French National Centre for Scientific Research in the Protist Collection of the Muséum National d’Histoire Naturelle, Paris, France, in the 1970s. We therefore used Chatton’s *C. elegans* slides to re-examine this parasite. Particular attention was paid to the tropho-tomont and tomite stages found in the renal appendages of *Sepia elegans*, as these stages were considered representative of the species [11] by Chatton and Lwoff in 1935 [4]. This work enabled us to formally designate a neohapantotype and paraneohapantotypes for the *C. elegans* species, from this slide collection.

Materials examined: Forty-eight slides corresponding to smears prepared by Chatton and Lwoff from renal appendages of *Sepia elegans* collected in Banyuls-sur-Mer, France [4], were deposited in the Protist Collection of the Muséum National d’Histoire Naturelle.

Redescription: The species description is based on the morphology of the tropho-tomont stage attached to the renal and pancreatic excretory epithelium, as previously proposed by Foettinger [11] then by Chatton and Lwoff [4]. Tropho-tomont body: thin, elongated, vermiform, length 30–1,400 μm, body average width 21.1 ± 3.3 μm (*N* = 132). Subpellicular macronucleus stained by haematoxylin, open and reticulated network of chromatin throughout the whole body (Fig. 1B).

Anterior end: Inflated, club-like (Figs. 1B–1D), terminated by a distinguishable apical papillum (Figs. 1C and 1D), attaching the tropho-tomonts to the renal tissues (Fig. 1A). Largest width of 53.2 ± 11.8 μm (*N* = 120).

Ciliature: Tropho-tomonts entirely covered by cilia (Fig. 1D); ciliature consisting of 14 kineties (Fig. 1E) originating from the apex, dextrally spiralled, directed antero-posteriorly continuously with no break (Fig. 1G).

Posterior end: Division by palintomy (Fig. 1F) with chains of primary segments (Fig. 1H), generating up to 24 protomites by fission (Fig. 1I). Protomites with condensed chromatin network (Fig. 1F). Tomites with ellipsoidal form (Fig. 1J) and size of 27.2 ± 1.7 μm by 17.8 ± 2.4 μm (*N* = 30). Presence of apotomites not confirmed.

## *Chromidina chattoni* Souidenne, Florent and Grellier n. sp. (Fig. 2)

urn:lsid:zoobank.org:act:A533901D-8325-4412-AF74-C63341DB03C7

**Figure 2.**
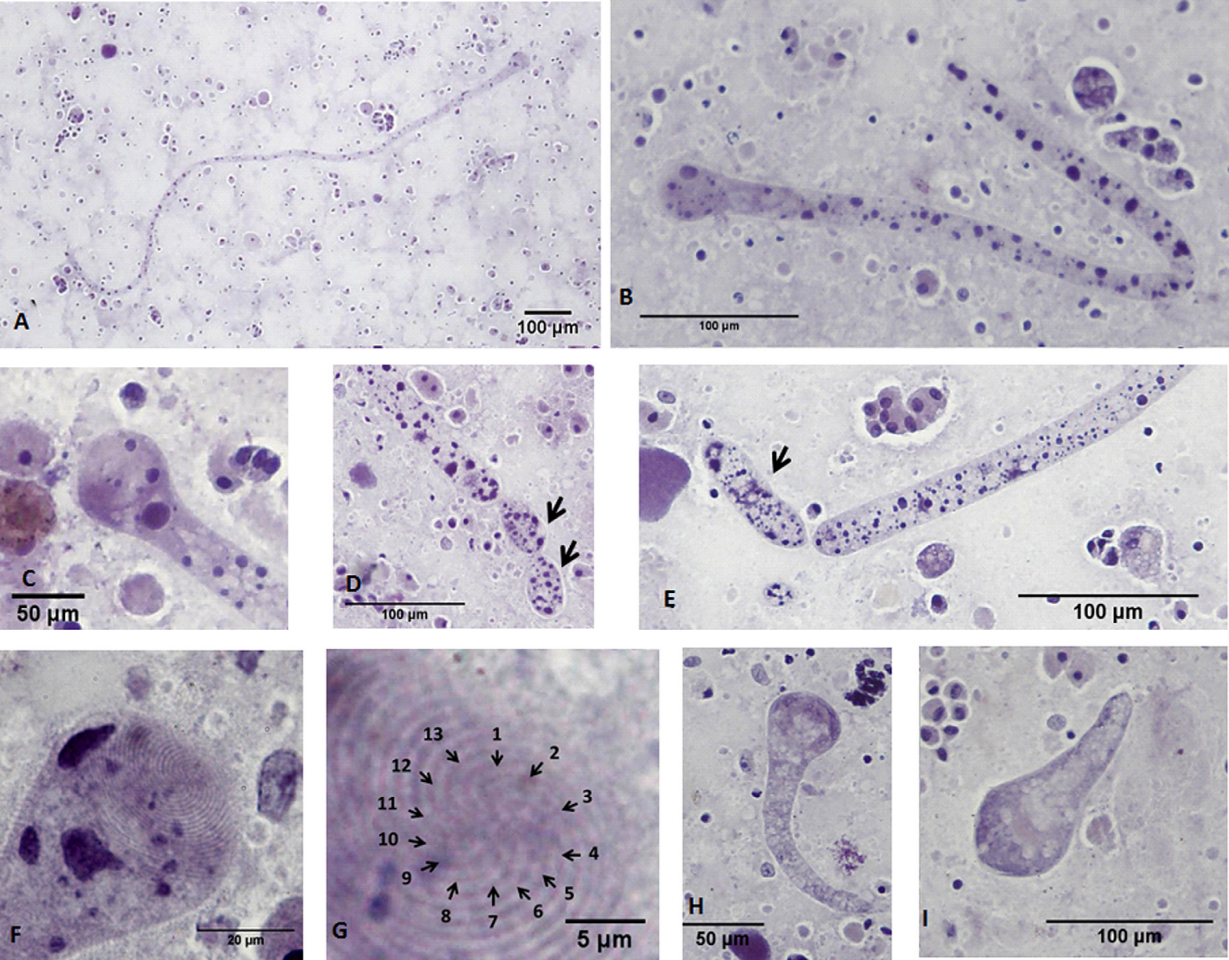
*Chromidina chattoni* Souidenne, Florent and Grellier n. sp.: morphology. (A, B) General views of tropho-tomonts. (C) Bulb-like head of tropho-tomont. (D, E) Palintomy with first generation of protomites (E) and second generation of protomites (D), arrows: protomites. (F) Bulb-like head of tropho-tomont. Note the presence of large and darkly-stained spots of chromatin associated with the head. (G) Ciliature consisting of 13 kineties (zoom of Fig. 2F). (H–I) Small forms of tropho-tomonts. Images were obtained from smears stained with haematoxylin. (A–I) Hapantotype and parahapantotypes.

Type host: *Loligo vulgaris* Lamarck, 1798.

Infection site: Renal appendages.

Type material: Hapantotype catalogued under No. MNHN-IR-2016-326 and parahapantotypes catalogued under Nos. MNHN-IR-MNHN-2016-327 to 341, haematoxylin-stained smears from the three infected *L. vulgaris* C21, C22 and C23, deposited in the Protist Collection of the Muséum National d’Histoire Naturelle, Paris, France.

Etymology: The species name was chosen in memory of the French biologist Edouard Chatton for his extensive and remarkable studies on apostomes.

Type locality: Off Tunis, Tunisia, Mediterranean Sea (36°49′9.11″ N, 10°18′22.49″ E).

DNA sequence: Partial sequences of 18S rDNA amplified from parasites isolated from the three infected *L. vulgaris* hosts deposited in the EMBL/GenBank/DDBJ database (Accession numbers: LT546660 (C21), LT546661 (C22) and LT546662 (C23)).

Prevalence: Sixty percent (three out of five specimens of *L. vulgaris* examined from off Tunis, Tunisia).

Authorship: Note that the authors of the new taxon are different from the authors of this paper, Article 50.1 and Recommendation 50A of the International Code of Zoological Nomenclature [21].

Description: Based on the morphology of the tropho-tomont stage. Parasite morphology identical on smears prepared from the three infected *L. vulgaris* hosts. Free swimming tropho-tomonts observed in renal fluids. Tropho-tomont body: Elongated and thin vermiform shape. Body width constant along body length (mean = 19.5 ± 3.7 μm, *N* = 60). Cytoplasm filled by darkly-stained islands of chromatin. Small tropho-tomonts weakly stained by haematoxylin, no clear network or spots of chromatin observed (Figs. 2H and 2I), suggesting degenerative forms rather than apotomite forms. Haematoxylin stain of tropho-tomonts revealing typical reticulated macronucleus of *Chromidina* [4], spreading throughout the entire cell body (Figs. 2B and 2E).

Anterior end (Fig. 2A): globular with regular width (mean = 40.8 ± 4.7 μm, *N* = 52). Typical bulb-like anterior end (Figs. 2A, 2B, 2C, 2F), but heterogeneous in length of 80–1,890 μm (mean = 657 ± 486 μm, *N* = 33). No distinguishable apical papillum observed. One to two large and darkly-stained spots of chromatin often associated with the bulb-like anterior end (Figs. 2B, 2C, 2F).

Ciliature: Consisting of 13 dextrally-spiralled kineties originating from the apex (Fig. 2G) and continuing uninterrupted on the entire cell body.

Posterior end: With typical *Chromidina* division segments that could generate either apotomites by monotony division or tomites by palintomy division (Figs. 2D and 2E).

Diagnosis: The *C. chattoni* n. sp. tropho-tomont has 13 kineties, which distinguishes it from other reported *Chromidina* species: 12 kineties for *C. cortezi* and the *Chromidina* sp. isolated from *Illex coindetti* in the Gulf of Mexico, and 14 for *C. elegans* [4] (Fig. 1E, Table 1). *C. coronata* has an unreported number of kineties but significantly differs from the other species by its bulbous anterior end covered by discernible superposed rows of dense and elongated cilia, presenting a crown-like aspect, which is the main identification feature for *C. coronata* (presence of a crown of cilia) [11, 17]. In addition, *C. chattoni* n. sp. differs from *C. elegans* by a bulb-like anterior end (Figs. 2B, 2C, 2F) versus an inflated club-like anterior end with a distinguishable apical papillum (Figs. 1C and 1D; [4]), and a narrower head (40.7 ± 4.7 μm versus 53.2 ± 11.8 μm, respectively). *C. cortezi* was described with a rounded anterior end with widths ranging from 22 to 48 μm [17].

*C. chattoni* n. sp. has been observed in *L. vulgaris* off Tunis which differs from *C. coronata* that has been observed in *Octopus vulgaris*, *Sepiola rondeleti*, *Illex coindetti*, *Eledone cirrhosa* and *Scaeurgus unicirrhus*, in the Mediterranean Sea and English Channel [4, 7, 10, 20], *C. cortezi* that has been observed in *Pterygioteuthis giardi* in the Gulfs of California and Mexico by Hochberg [17, 20], and *Chromidina* sp. described by Landers [25], that has been isolated from *Illex coindetti* in the Gulf of Mexico.

## *Chromidina* sp. S50 from *Sepia officinalis* (Fig. 3)

Host: *Sepia officinalis* Linnaeus, 1758.

**Figure 3.**
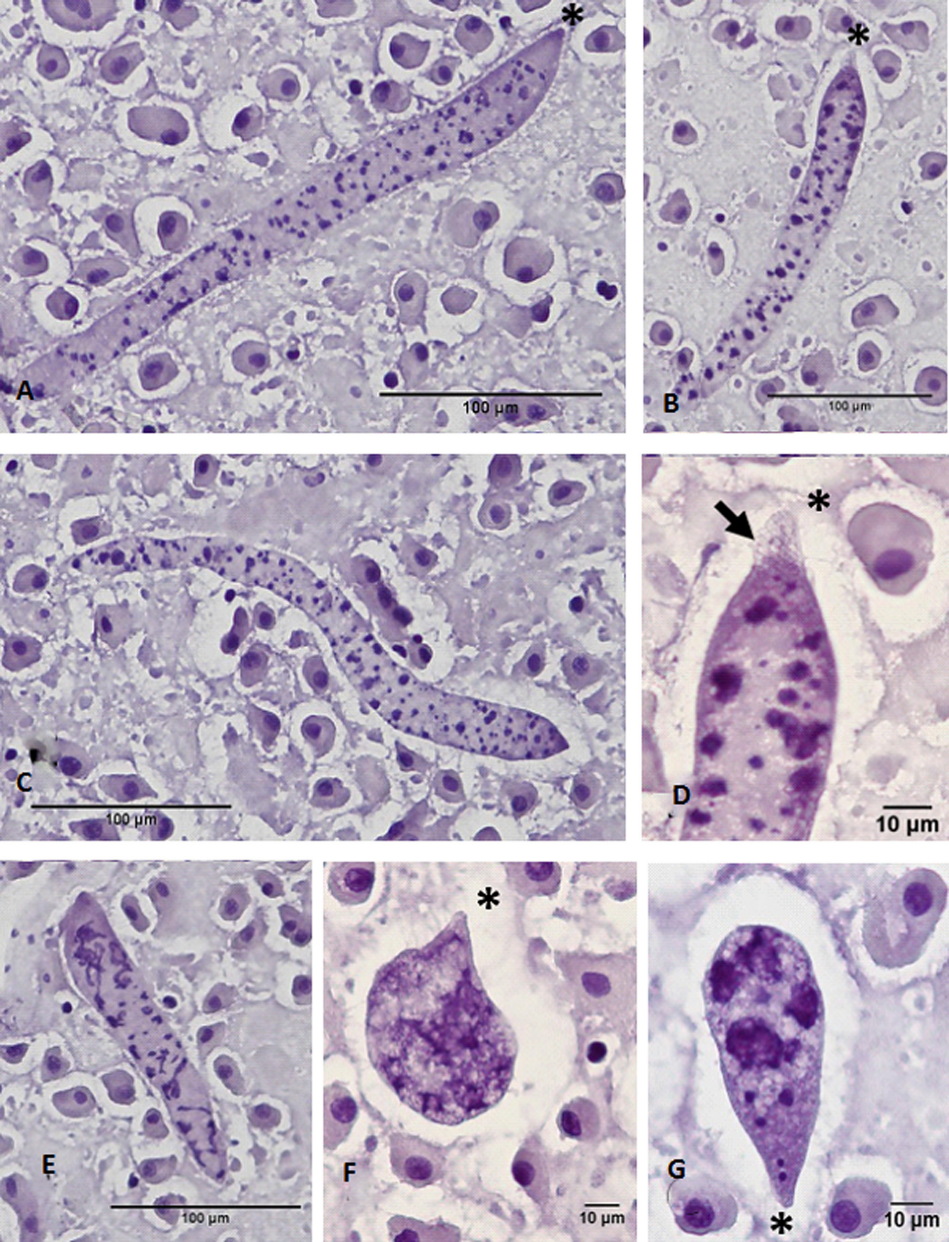
*Chromidina* sp. S50: morphology. (A–C, E) Tropho-tomonts: anterior end has a conical shape terminated by an apical papillum. (D) Enlargement of Figure 3B showing the apical papillum and the ciliature consisting of spiralled kineties originating from at the apex (arrow). (F, G) Degenerative-like or cyst-like forms. *: apical papillum.

Infection site: Renal appendages.

Material: Haematoxylin-stained smears from the infected *S. officinalis* S50, deposited in the Protist Collection of the Muséum National d’Histoire Naturelle, under No. MNHN-IR-2016-108.

Locality: Off Tunis, Tunisia, Mediterranean Sea (36°49′9.11″ N, 10°18′22.49″ E).

DNA sequence: Partial sequences of 18S rDNA amplified from parasites isolated from *S. officinalis* host S50 deposited in the EMBL/GenBank/DDBJ database (Accession number LT546663).

Prevalence: 2.6% (one out of 38 specimens of *S. officinalis* examined from off Tunis, Tunisia).

Description: Tropho-tomonts from only one *S. officinalis* out of 38 specimens examined; infection at low level; host co-infected by the dicyemid *Pseudicyema truncatum* (data not shown).

Tropho-tomonts: Vermiform (Fig. 3A), length of 99.6–481.1 μm (mean = 259.1 ± 93.0 μm, *N* = 38), average body width of 23.4 ± 4.9 μm (*N* = 117). Smallest tropho-tomonts with stockier and wider body (Fig. 3E). Small and rounded forms also observed, suggesting either degenerative forms or encystment process (Figs. 3F and 3G). Cytoplasm filled by darkly-stained islands of chromatin. Typical reticulated macronucleus of *Chromidina* spp. revealed by haematoxylin stain [4], spreading throughout the entire cell body.

Anterior end: Majority of parasites with conical shape terminated by a pronounced apical papillum attaching the parasite to the renal epithelium; width of the anterior end similar to that of the body (Figs. 3A–3E). Slight inflation of the head observed in a few parasites.

Ciliature: Consisting of dextrally-spiralled kineties originating from the apex and continuing on the cell body (Fig. 3D). Number of kineties not determined due to unfavourable positioning of tropho-tomonts on smears. Posterior end: long tropho-tomonts with narrower posterior end than anterior part (Figs. 3A and 3C). No division (monotomy or palintomy) observed.

Remarks: The absence of dividing stages and the impossibility of determining the number of kineties limit the comparison with the other *Chromidina* species. The absence of a crown of cilia differentiates it from *C. coronata* and this type of tropho-tomont morphology with a conical head was not observed for *C. elegans*. Hochberg [17, 20] described apotomites of *C. cortezi* with similar morphology on the renal appendages of the squid *Pterigioteuthis giardi* fished in the Gulfs of California and Mexico. Apotomites are large single buds produced by a monotonic process from tropho-tomonts. When detached, they differentiate into a second generation of tropho-tomonts that colonise the renal appendages. It is suggested that stress conditions such as a shortage of nutrients or of essential metabolites might result in the production of apotomites instead of tomites [17]. The presence of degenerative or cyst-like forms (Figs. 3F and 3G) in our preparations supports such stress conditions. Furthermore, co-infection with dicyemids indicates that the infection by this *Chromidina* sp. in *S. officinalis* S50 reaches its final stage. The *Chromidina* sp. population is progressively replaced by dicyemids, competing for the same ecological habitat, the renal appendages, when the host changes from a pelagic to a benthic life cycle [13]. *Pterigioteuthis giardi* was originally described from the Mediterranean Sea but is now known to be nearly cosmopolitan [17]. Whether the *Chromidina* sp. observed in our preparations corresponds to *C. cortezi* needs to be clarified and requires further investigation. In any case, this is the first description of a *Chromidina* sp. infecting *Sepia officinalis*.

Table 1 summarises the specific characters of each *Chromidina* sp. described to date including *Chromidina chattoni* n. sp. from *L. vulgaris* and *Chromidina* sp. S50 from *S. officinalis,* both identified in the current study.

## Phylogenetic analysis

**Figure 4.**
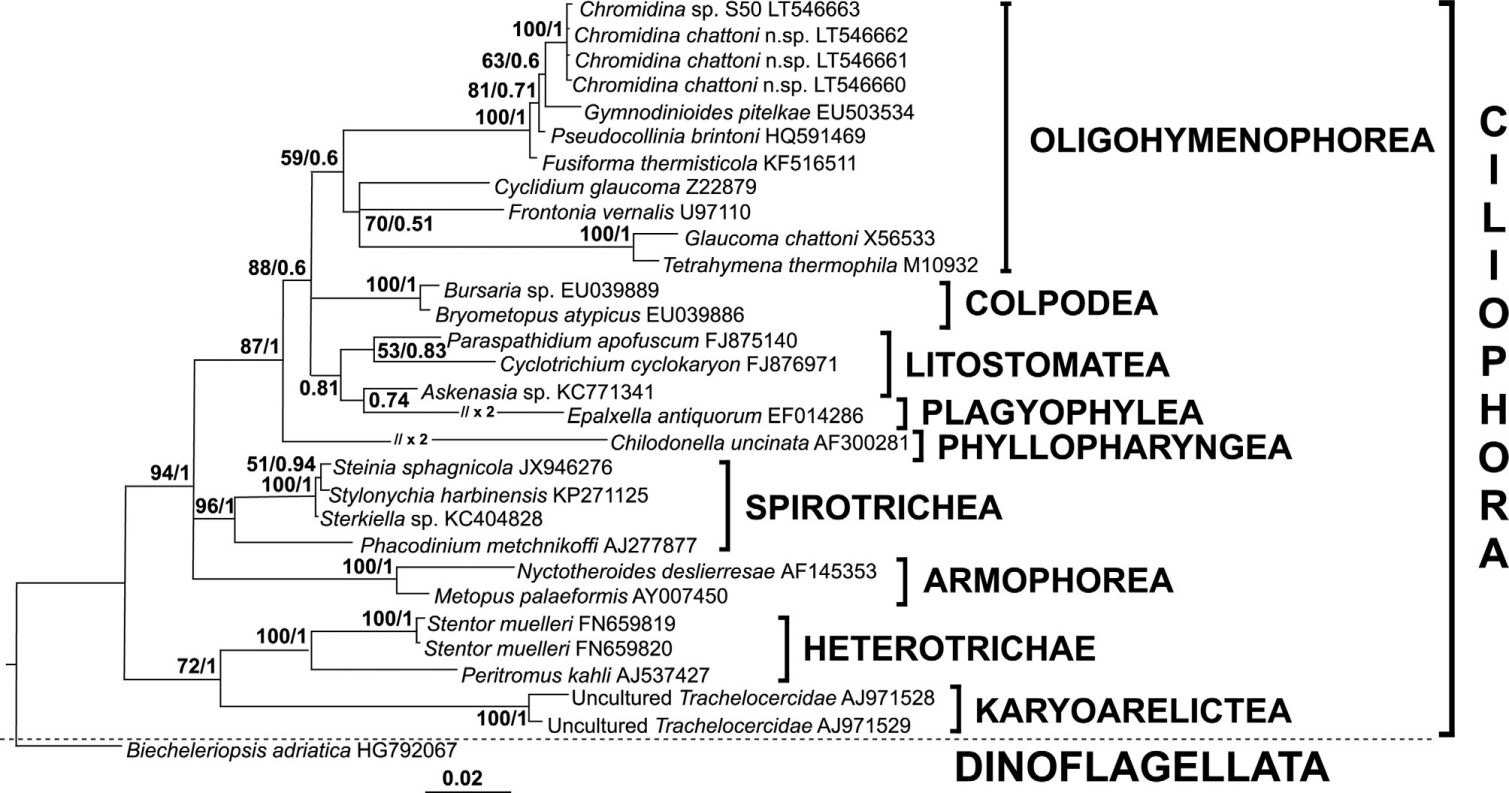
Phylogenetic position of *Chromidina* spp. within Ciliophora. This phylogenetic tree, rooted on a dinoflagellate rDNA sequence, was inferred from 30 small subunit (SSU) 18S rDNA sequences corresponding to the four *Chromidina* specimens identified in this study, 25 representatives of the major classes of the phylum Ciliophora, and one Dinoflagellata sequence taken as the outgroup. There were a total of 1,157 positions in the final dataset. Both the Maximum Likelihood (ML) method and Bayesian inferences, based on the general time-reversible +G +I model [28], were conducted and yielded similar topologies; the currently presented topology was obtained by the Bayesian inference. The tree is drawn to scale, with branch lengths measured in number of substitutions per site. Some branches were shortened by multiples of the length of substitutions/site scale bar (Plagyophylea, Phyllopharyngea). Numbers at the branches denote ML bootstrap percentage, from 1,000 resampling (first value) and Bayesian posterior probabilities (second value).

Positive PCRs were obtained for each of the four *Chromidina* specimens collected from the infected cephalopod hosts, using universal and specific primers designed to amplify a portion of the *Chromidina* 18S rDNA locus. Gene sequencing and assemblage enabled us to determine, for the first time, the partial 18S rDNA sequences for these parasite ciliates. Blast analyses revealed that the highest homology scores were obtained with known Apostomatia 18S rDNA sequences, in particular with species belonging to the genus *Pseudocollinia* [14, 27]. A multiple alignment was built using these four *Chromidina* spp. sequences and a representative selection of Apostomatia to compute evolutionary distances (Table 2). Bayesian and Maximum Likelihood phylogenetic trees were also constructed using a selection of species belonging to several Ciliophora classes (Fig. 4).

**Table 2.** Genetic distances (%) between sequences of representative Apostomatia.

	1	2	3	4	5	6	7	8	9	10	11	12	13
1 *Chromidina chattoni* LT546660 (C21)													
2 *Chromidina chatton*i LT546661 (C22)	0.00												
3 *Chromidina chattoni* LT546662 (C23)	0.07	0.07											
4 *Chromidina* sp. S50 LT546663 (S50)	0.20	0.20	0.14										
5 *Pseudocollinia brintoni* HQ591470	3.99	3.99	3.93	4.06									
6 *Pseudocollinia similis* HQ591485	4.06	4.06	3.99	4.13	0.34								
7 *Pseudocollinia beringensis* HQ591476	4.13	4.13	4.06	4.20	0.27	0.07							
8 *Pseudocollinia oregonensis* HQ591473	4.13	4.13	4.06	4.20	0.34	0.47	0.47						
9 *Fusiforma themisticola* KF516511	4.81	4.81	4.74	4.74	1.90	1.83	1.90	2.03					
10 *Gymnodinioides pitelkae* EU503534	5.96	5.96	5.96	6.09	4.13	4.27	4.33	4.13	4.33				
11 *Hyalophysa lwoffi* EU503538	6.09	6.09	6.16	6.30	4.54	4.47	4.54	4.47	4.54	3.45			
12 *Vampyrophrya pelagica* EU503539	6.09	6.09	6.03	6.16	4.67	4.67	4.67	4.40	4.60	3.32	2.44		
13 *Hyalophysa chattoni* EU503536	6.23	6.23	6.16	6.16	5.15	5.15	5.21	5.01	5.21	4.13	1.76	2.57	
14 *Gymnodinioides* sp. EU503535	6.36	6.36	6.36	6.50	4.40	4.54	4.54	4.27	4.20	3.05	2.17	1.35	2.78

The numbers of base differences per site between sequences are shown as percentages. The analysis involved 14 nucleotide sequences. All ambiguous positions were removed for each sequence pair. There were a total of 1,477 positions in the final dataset.

The three sequences obtained for *Chromidina* species infecting *L. vulgaris* hosts were highly similar to each other and very close to the sequence obtained for the *Chromidina* sp. S50 infecting *S. officinalis*, with a genetic divergence of less than 0.2% (Table 2). The closest relatives of these *Chromidina* sp. sequences were those of *Pseudocollinia brintoni* (e.g. HQ591470, [14]), *Pseudocollinia similis* (e.g. HQ591485, [27]) *Pseudocollinia beringensis* (e.g. HQ591476, [27]) and *Pseudocollinia oregonensis* (e.g. HQ591473, [27]) with a genetic divergence of 3.9–4.2%, then *Fusiforma themisticola* (KF516511, [3]) with a genetic divergence of 4.7–4.8%. *Gymnodinioides pitelkae* (EU503534, [5]), *Hyalophysa lwoffi* (EU503538, [5]) and *Vampyrophrya pelagica* (EU503539, [5]), and *Hyalophysa chattoni* (e.g. EU503536.1, [5]) were found with a genetic divergence of 5.9–6.2% and *Gymnodinioides* sp. (EU503535.1, [5]) of ~6.4% (Table 2). These values reveal the rather low divergence between these species, for this molecular marker, as previously observed by Lynn for other Apostomatia [27]. *Pseudocollinia* spp., parasitoid apostomes of krill, currently appear the species most closely related to *Chromidina* spp. The phylogenetic analysis using a dinoflagellate sequence as an outgroup revealed that, within the Ciliophora phylum, these *Chromidina* spp. sequences remained grouped with the Apostomatia (*Chromidina*, *Gymnodinioides*, *Pseudocollinia*, *Fusiforma*) within the Oligohymenophorea class, with strong bootstrap values (Maximum Likelihood analysis) and posterior probability values (Bayesian analysis) (Fig. 4).

## Conflict of interest

The Editor-in-Chief of Parasite is one of the authors of this manuscript. COPE (the Committee on Publication Ethics, http://publicationethics.org), to which Parasite adheres, advises special treatment in these cases. In this case, the final stage of the peer review process was handled by an Invited Editor, Jérôme Depaquit.
